# Identification of Lactic Acid Bacteria From the Crust and Inner Part of Artisanally Produced Mihaliç Cheese Sold With Salty and Low Salty Label by Using MALDI‐TOF‐MS and 16S rDNA Sequencing

**DOI:** 10.1002/fsn3.71489

**Published:** 2026-02-01

**Authors:** Ergün Ayanoğlu, Hakan Tavşanlı, Recep Cibik

**Affiliations:** ^1^ Bursa Uludag University Karacabey Meslek Yüksek Okulu Bursa Türkiye; ^2^ Faculty of Veterinary Medicine, Department of Food Hygiene and Technology, Çağış Campus Balıkesir University Balıkesir Türkiye; ^3^ Faculty of Veterinary Medicine, Department of Food Hygiene and Technology, Görükle Campus Bursa Uludag University Bursa Türkiye

**Keywords:** inner part crust and, lactic acid bacteria, Lactobacillus spp., low salty cheese salty and, mihaliç cheese, *Streptococcus gallolyticus*, *Streptococcus infantarius*

## Abstract

Mihaliç is a traditional cheese manufactured using artisanal methods without adding starter cultures from a mixture of sheep's and cow's milk. The indigenous microbiota originating from raw milk and the surrounding environment, which adapt to the ripening conditions, play a crucial role in developing the artisanal cheeses' characteristic flavor, aroma, and texture. In this study, the lactic acid bacteria in both the crust and the inner part of Mihaliç sold under the label of Salty Mihaliç Cheese (SMC) and Low‐Salty Mihaliç Cheese (LSMC) were determined using MALDI‐TOF MS analysis and 16S rDNA sequencing. The enumeration of Streptococcaceae on M17 agar revealed approximately a 2‐log higher count compared to *Lactobacillus* and *Enterococcus* populations. Among the isolates recovered from M17 medium, *Lactococcus lactis* was identified as the predominant species, comprising 43.7% of the total. Notably, consistent with observations in many other artisanal raw milk cheeses, *Streptococcus gallolyticus* subsp. *macedonicus* and *Streptococcus infantarius* subsp. *infantarius* were also detected at high frequencies, with 41.2% and 7.5% prevalence rates, respectively. Among the enterococci, *Enterococcus faecium* and *Enterococcus faecalis* were the most frequently isolated species, accounting for 43.5% and 42.2% of the isolates, respectively. Regarding the distribution of lactobacilli, *Lacticaseibacillus casei/paracasei* and *Limosilactobacillus fermentum* emerged as the dominant species, exhibiting prevalence rates of 47.1% and 35.2%, respectively. Meanwhile, comparative analysis between SMC and LSMC cheese samples showed that *Lim. fermentum* was more abundant in SMC samples (55.1%), whereas *Lcb. paracasei/casei* was dominant in LSMC samples (55.9%). Furthermore, both *Lim. fermentum* and *Lcb. rhamnosus* were more frequently recovered from the inner part of the cheese matrix. These findings highlight the complex and diverse lactic microbiota of Mihaliç cheese, which is likely to play a significant role in the development of its unique flavor and textural characteristics.

## Graphical abstract

Our results indicate that streptococci were predominant, particularly in the crust, and that *Lacticaseibacillus paracasei*/*casei* and *Limosilactobacillus fermentum* were the most common lactobacilli, with notable differences between SMC and LSMC samples. Furthermore, the presence of *Streptococcus gallolyticus* ssp. *macedonicus* and *Str. infantarius* ssp. infantarius as dominant species aligns with patterns seen in other raw milk cheeses. This is the first study to characterize the lactic microbiota of Mihalic cheese using this dual‐method approach.

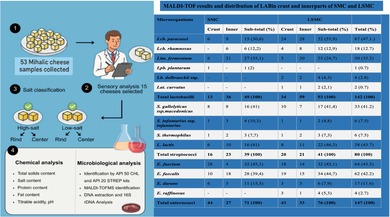

## 1 Introduction

In Türkiye, there are more than 250 different cheese varieties, and most of them are produced in rural areas according to traditional manufacturing techniques without the addition of starter culture (Özcan and Kurdal 2012). Mihaliç cheese is one of the traditionally produced artisanal cheeses around Bursa and Balikesir provinces in north‐western Türkiye (Aday and Karagul Yuceer 2014). It has been manufactured for nearly 200 years in the region and takes third place in Türkiye's cheese marketing in terms of production (Hayaloglu et al. 2008). Raw sheep's milk (Kivircik sheep's) or a mixture of sheep's, goat's, and/or cow's milk is used in Mihalic fabrication (Akın 1997). Previously, Mihaliç was produced only between April and June due to the availability of sheep milk; however, currently, its production continues throughout the year, mostly in small‐scale dairies.

Mihaliç is a brine‐ripened hard‐type cheese with an ovoid shape and approximately 20–25 cm diameter. It is characterized by an outer crust that is 8–10 mm thick and a relatively soft and yellowish inner part. The inner part contains several eyes of 3–4 mm in diameter; however, their numbers and sizes decrease towards the crust part (Bulut 2006). The ripening process is achieved in wooden barrels containing 18%–20% brine for 4 months between 15°C and 25°C (Kamber 2008; Aday and Karagul Yuceer 2014). Nowadays, some manufacturers produce relatively lower salt containing Mihaliç as well. Cheese curds, after being kept in 18%–20% brine for two days, are then taken in 16% brine for the rest of the ripening period (Evrensel and Yıldız 1997; Özcan 2000; Üçüncü 2004). Since Mihaliç is produced artisanally without adding starter cultures, competitive microorganisms from raw milk and surroundings account for the characteristic aroma of formation over‐ripening. Microorganisms from the raw milk used, production steps, ripening conditions and environmental factors affect the microbial dynamics, causing the formation of the dominant flora specific to the cheese and, thus, the formation of typical aroma, flavor and textural qualities. Potential starter and non‐starter lactic acid bacteria (NSLAB) found in artisanal cheeses support the formation of metabolites such as organic acids, volatile short‐chain fatty acids, alcohols, ethers, etc., which are responsible for the aroma, flavor and textural structure in cheeses, through acidification, proteolysis and lipolysis steps (Nalepa and Markiewicz 2022).

Matrix‐assisted laser desorption ionization‐time of flight mass spectrometry (MALDI‐TOF MS) is a rapid, cost‐effective, and highly reliable analytical tool for characterizing diverse microorganisms from different environments, including food. With this technique, microbial colonies can be identified at the species level with high accuracy without requiring a supplementary pre‐treatment (Gantzias et al. 2020). The accuracy and validity of the technique increase as the microbial protein database includes bacteria, fungi, and viruses (Akimowicz and Bucka‐Kolendo 2020).

The lactic acid bacteria (LAB) and associated microbial communities responsible for the development of desirable aromatic properties and the characteristic openings within the interior of Mihaliç cheese have not yet been fully elucidated. In recent years, there has been growing interest in isolating and characterizing bacterial strains with potential applications in cheese manufacturing, particularly from traditionally produced varieties (Uymaz et al. 2019). Cheeses such as Mihaliç, which are produced using artisanal practices without the addition of starter cultures, represent valuable reservoirs for the isolation of LAB strains that may contribute to the development of distinctive flavors, aromas, and textures preferred by consumers. In this study, we aimed to isolate and identify the autochthonous LAB species present in Mihaliç cheese samples ripened and marketed under different salt concentrations (high and low). Bacterial species and subspecies were identified using Matrix‐Assisted Laser Desorption/Ionization Time‐of‐Flight Mass Spectrometry (MALDI‐TOF MS) and 16S rDNA gene sequencing analysis.

## 2 Material and Methods

### 2.1 Cheese Samples

**FIGURE 1:**
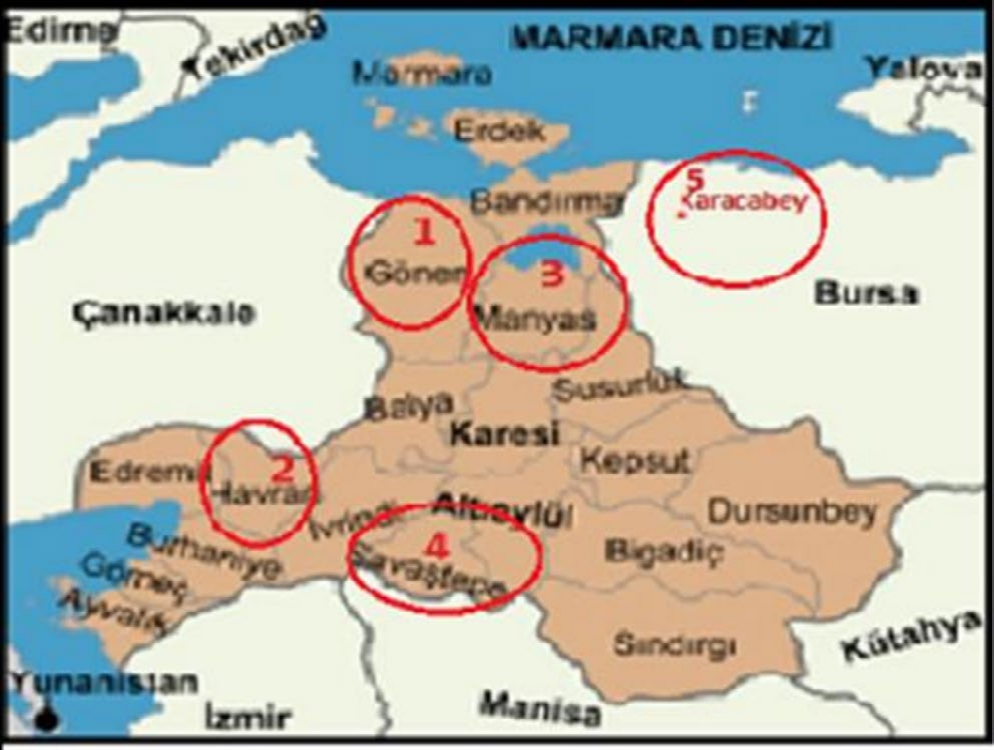
Locations where Mihaliç cheese was collected.

A total of 53 Mihaliç cheeses produced according to traditional techniques and sold under different brand names were collected from producers, local markets and public bazaars in Balikesir and Bursa provinces (Gönen, Manyas; Savaştepe, Havran, Karacabey districts) in Marmara Region and transported to the laboratory at 4°C for analyses. This region is known for the highest production ratio of Mihaliç. Districts where the samples were collected are shown in Figure 1.

### 2.2 Sensory Evaluation of Cheeses

The hedonic scale was used to determine the organoleptic qualities of collected cheeses (Drake 2007). Ten panelists with a certain extent of specialization in characteristic properties of Mihaliç cheese evaluated the samples. Each of the color, appearance, texture, odor, and taste parameters was separately scored between 1 and 9 (1—extraordinarily bad, 2—very bad, 3—bad, 4—slightly bad, 5—average, 6—slightly good, 7—good, 8—very good, 9—extraordinarily good). A higher score represents a quality close to perfect (5). Organoleptic evaluation results conducted us to select 15 samples from each of saline (SMC) and low saline Mihaliç cheeses (LSMC) according to their relatively high score. Further analysis and microbial isolations were performed on these selected cheese samples.

### 2.3 Microbiological Analyzes

The crusts and the inner parts of the cheese were separately analyzed for microbiological evaluations. 10 g portions of the sample were mixed with 90 mL MRD (Maximum Recovery Diluent, LabM103‐A) and homogenized. After homogenization, serial dilutions were prepared up to 10^−8^ dilutions. Appropriate dilutions were plated onto MRS Agar (Isolab, LAB223) for lactobacilli, M17 Agar for streptococci (Isolab, LAB092), and Kanamycin Esculin Azide Agar (Isolab, LAB106) for enterococci. Plates were then incubated at 37°C in anaerobic conditions for lactobacilli and aerobic conditions for streptococci and enterococci (Mathara et al. 2004). Three to five unique and typical colonies growing on plates were selected for their morphologic appearance, Gram staining, and catalase activity (Bactident catalase, Sigma) before further identification.

### 2.4 MALDI‐TOFMS Identification

The selected and pre‐characterized colonies were identified using MALDI‐TOF MS and the VITEK MS system (BioMérieux, France). Samples were taken with a sterile pipette tip from the colony's surface and transferred directly onto the sample spots on the target slide. The deposited bacteria were overlaid with 1 μL of CHCA matrix (a saturated solution of α‐cyano‐4‐hydroxycinnamic acid in 50% acetonitrile‐2.5% trifluoroacetic acid) and air dried at room temperature to allow co‐crystallization with the experimental sample (Nacef et al. 2017). The target slide was loaded into the VITEK‐MS system to obtain the bacterial cell protein profile, and then the acquired mass spectra were compared to those of known spectra loaded into the database. A confidence value > 90% was considered for the identification of microorganisms; however, most of our isolates had a confidence value of 99.9%.

### 2.5 DNA Extraction and 16S rDNA Analysis

For 16S rDNA gene sequencing, the total DNA from overnight cultures of 238 isolates was extracted using a Highpure Template DNA Isolation Kit (Roche Diagnostics, Mannheim, Germany). Most of the isolates were from this study, while some (50) were lab isolates from Mihaliç cheeses at the preliminary part of the study. The DNA concentration was evaluated spectrophotometrically by NanoDrop ND2000 spectrophotometer (Thermo Scientific, USA). DNA was then stored at −20°C until used as templates for amplification.

PCR amplification of 16S rDNA was performed using the forward primer (27‐F) 5′‐AGAGTT TGA TCC TGG CTC AG‐3′ and reverse primer (1492‐R) 5′‐TAC GGT TAC CTT GTT ACG ACT T‐3′ (Peace et al. 1994). PCR mixture contained 1 μL of template DNA, 10 μL 5× PCR buffer, 0.4 μL dNTPs, 1 μL of 20 mM primers FP and RP, 0.25 μL 5 U Taq polymerase and sterile H_2_O up to 50 μL of total volume. The reaction was performed with the following program: 95°C for 2 min, 35 cycles of 95°C for 20 s, 55°C for 40 s, and 72°C for 50 s with a final extension of 72°C for 5 min. The PCR products were purified using a GeneJET PCR purification kit (Thermo Scientific, USA) as per the manufacturer's instructions and analyzed on 1% agarose gel electrophoresis. Forward and reverse sequencing reactions were performed with BigDye Terminator cycle sequencing ready reaction kit reagents on an ABI Prism 310 or 377 DNA sequencer (Applied Biosystems, Foster City, CA, USA) by standard protocols. The sequence data were assembled, edited, and identified via BLAST search in the GenBank database to identify the closest available reference sequences in the complete National Center for Biotechnology Information (NCBI) nucleotide collection. Accordingly, the accession numbers of the 238 isolates submitted to NCBI are registered with MK744064 through MK744073 and MK962023 through MK962106.

### 2.6 Chemical Analyzes

The crust and inner parts of the cheese sample were analyzed for chemical evaluation. Total dry matter was determined as suggested by (TS EN ISO 5534 2006), and salt content was determined according to the Mohr method (ISO 1738 2004). Kjeldahl method was used to determine protein content (AOAC 2001–6). Fat content was determined as suggested by (TS 3046 1978), and the acidity was determined titrimetrically according to (TS 591 2013).

### 2.7 Statistical Analysis

Data analysis was done using IBM Statistical Package for Social Sciences (SPSS) software version 20.0 for Windows (SPSS Inc., Chicago, IL, USA). The sensory evaluation data of cheeses was analyzed using the Kolmogorov–Smirnov test and found to be normally distributed; therefore, Student's *t*‐test was applied for pairwise comparisons. For the chemical and microbial analyses of the cheese samples, one‐way analysis of variance (ANOVA) was conducted to determine differences among groups, and significant differences were identified using Tukey's multiple comparison test (*p* < 0.05).

## 3 Results

Data regarding the organoleptic features of cheeses tested by panelists are presented in Table 1. The average organoleptic quality scores of SMC and LSMC samples were between 5.80 and 5.85. The color quality score of SMC samples (6.16) is higher than LSMC's (5.44). On the other hand, the different parameters, such as appearance, texture, odor, and taste quality, of the LSMC samples were higher than those of the SMC samples. Data related to the mean values of dry matter, fat in dry matter, pH, acidity, and salt content in the crust and inner parts of SMCs and LSMCs is presented in Table 2. As expected, a considerable difference was noted for the salt content of SMC and LSMC cheeses; however, no statistically significant differences were determined between the crusts and inner parts of the cheeses in terms of other chemical properties (*p* > 0.05).

**TABLE 1:** The organoleptic evaluation of cheeses.

Parameter	SMC	LSMC
Color	6.16 ± 0.76^a^	5.44 ± 1.26^b^
Outlook	5.63 ± 0.5	5.75 ± 0.96
Texture	5.83 ± 0.39^a^	6.2 ± 0.72^b^
Smell	5.64 ± 0.09	5.88 ± 0.08
Taste	5.76 ± 0.79	6 ± 0.3
General	5.8 ± 0.35	5.85 ± 0.30

*Note:* Different letters in the same line indicate statistically significant difference (*p* < 0.05).

**TABLE 2:** Physical and chemical analysis of cheeses.

Analysis	SMC		LSMC	
	Crust	Inner	Crust	Inner
Dry matter	59.47 ± 1.41	57.80 ± 1.28	58.44 ± 1.92	56.47 ± 1.36
Fat in dry matter	44.39 ± 0.79^a^	45.14 ± 1.1^a^	49.89 ± 1.39^b^	48.16 ± 1.38^b^
Protein	21.20 ± 0.25	22.12 ± 1.02	20.85 ± 0.85	21.26 ± 0.29
pH	5.26 ± 0.88^a^	5.28 ± 0.06^a^	5.64 ± 0.16^b^	5.61 ± 0.55^b^
Acidity (%LA)	1.64 ± 1.93^a^	1.54 ± 0.14^a^	1.24 ± 0.21^b^	1.26 ± 0.25^b^
Salinity	6.02 ± 1.32^a^	6.31 ± 0.42^b^	4.48 ± 0.29^c^	4.49 ± 0.32^c^

*Note:* Different letters in the same line indicate statistically significant difference (*p* < 0.05).

As presented in Table 3, the mean counts of Streptococcaceae, lactobacilli, and enterococci differed between SMC and LSMC groups, as well as between the crust and inner parts. In the SMC group, the Streptococcaceae population was approximately 1 log unit higher than that of lactobacilli and enterococci. On the crust, Streptococcaceae levels were nearly 2 log units greater than those of other bacterial groups, suggesting a stronger adaptation to the maturation conditions of Mihaliç cheese. Lactobacilli counts were about 1 log unit higher in LSMC and in the inner parts compared to SMC and crust samples, respectively (*p* < 0.05), indicating their higher sensitivity to salinity. Similarly, enterococci were also affected by salt content, as their counts were nearly 1 log unit lower in the crust compared to the inner parts (*p* < 0.05).

**TABLE 3:** The LAB count in SMC and LSMC; and the crust and inner parts of Mihaliç cheese.

Sample	*Lactobacillus* spp.	Streptococccaceae	*Enterococcus* spp.
SMC	6.2 ± 0.52^a^	7.1 ± 0.47	6.09 ± 0.19
LSMC	7.06 ± 0.44^b^	7.27 ± 0.12	6.14 ± 0.28
Crust	5.30 ± 0.52^c^	7.00 ± 0.27	5.26 ± 0.31^b^
Inner	6.78 ± 0.29^d^	6.78 ± 0.32	6.31 ± 0.18^a^

*Note:* Different letters for each of SMC/LSMC or Crust/Inner columns indicate statistically significant difference (*p* < 0.05).

MALDI‐TOF MS analysis of 369 selected colonies representing lactobacilli (142), Streptococcaceae (80), and enterococci (147) is presented in Table 4. Among lactobacilli, *Lcb. casei/paracasei* (47.1%) and *Lim. fermentum* (35.2%) were the predominant species. *Lim. fermentum* was more abundant in the SMC group (55.1%), whereas *Lcb. casei/paracasei* dominated the LSMC group. In addition, *Lim. fermentum* was the most frequently identified species from the inner part of the cheeses. Similarly, *Lcb. rhamnosus* was more prevalent in the inner part, with an overall proportion of 12.7%. Although not dominant, *Lb. delbrueckii* spp., *Latilactobacillus curvatus*, *Lentilactobacillus parabuchneri*, and *Lactiplantibacillus plantarum* were detected at lower frequencies ranging from 1% to 4%.

**TABLE 4:** MALDI‐TOF results and distribution of LAB in crust and inner parts of SMC and LSMC.

Microorganisms	SMC			LSMC			Total (%)
	Crust	Inner	Sub‐total (%)	Crust	Inner	Sub‐total (%)	
*Lcb. casei/paracasei*	6	9	15 (30.6)	24	28	52 (55.9)	67 (45.7)
*Lcb. rhamnosus*	—	6	6 (12.2)	4	8	12 (12.9)	18 (12.2)
*Lim. fermentum*	6	21	27 (55.1)	3	20	23 (24.7)	50 (34)
*Lpb. plantarum*	1	—	1 (2)	—	—	—	1 (0.7)
*Lb. delbrueckii* ssp.	—	—	—	2	2	4 (4.3)	4 (2.7)
*Lat. curvatus*	—	—	—	1	1	2 (2.1)	2 (1.3)
*Len. parabuchneri*	2	—	2 (40)	2	1	3 (60)	5 (3.4)
Total lactobacilli	15	36	51 (100)	36	60	96 (100)	147 (100)
*S. gallolyticus* ssp. *macedonicus*	8	8	16 (41)	10	7	17 (41.4)	33 (41.2)
*S. infantarius* ssp. *infantarius*	1	3	4 (10.3)	1	1	2 (4.8)	6 (7.5)
*S. thermophilus*	1	2	3 (7.7)	1	2	3 (7.3)	6 (7.5)
*L. lactis*	6	10	16 (41)	8	11	19 (46.3)	35 (43.7)
Total Streptococcaceae	16	23	39 (100)	20	21	41 (100)	80 (100)
*E. faecium*	28	4	32 (45.1)	18	14	32 (42.1)	64 (43.5)
*E. faecalis*	10	18	28 (39.4)	19	15	34 (44.7)	62 (42.2)
*E. durans*	6	5	11 (15.5)	3	3	6 (7.9)	17 (11.6)
*E. raffinosus*	—	—	—	3	1	4 (5.3)	4 (2.7)
Total enterococci	44	27	71 (100)	43	33	76 (100)	147 (100)

Within Streptococcaceae, *L. lactis* was the predominant species (43.7%), consistent with its role as the major component of the dominant microbiota in many cheese varieties. Interestingly, similar to other artisanal raw milk cheeses, *S. gallolyticus* ssp. *macedonicus* (41.2%) and *S. infantarius* ssp. *infantarius* (7.5%) represented the main streptococcal taxa in Mihaliç cheese. Regarding enterococci, *E. faecium* and *E. faecalis* together accounted for nearly 85% of isolates, while *E. durans* and *E. raffinosus* were detected at lower proportions.

MALDI‐TOF‐MS's effectiveness for identifying tested bacteria was compared with 16S rDNA sequencing. It is worth noting that there was no remarkable inconsistency between the techniques used; however, as shown in Table 5, some isolates of *Lcb. paracasei/casei* and *S. infantarius* ssp. *infantarius* have been named differently by 16S rDNA sequencing, suggesting that additional identification tools may be required to identify closely related species/subspecies precisely.

**TABLE 5:** Comparison of MALDI‐TOF‐MS and 16S rDNA sequencing results.

MALDI‐TOF‐MS identification	Number of isolates	16S rDNA sequencing	Number of isolates
*Lcb. paracasei/casei*	85	*Lcb. paracasei/casei*	66
		*Lcb. rhamnosus*	16
		*Loig. coryniformis*	3
*Lcb. rhamnosus*	13	*Lcb. rhamnosus*	13
*Lim. fermentum*	80	*Lim. fermentum*	77
		N.I.	3
*Lpb. plantarum/pentosus* *Lat. curvatus*	8	*Lpb. plantarum* N.I.	71
	6	*Lat. curvatus*	3
		N.I.	3
*Lb. delbrueckii* spp.	2	*Lb. delbrueckii* spp. *lactis*	2
*Len. parabuchneri*	5	*Len. parabuchneri*	2
		N.I.	3
*S. infantarius* ssp. *infantarius*	10	*S. infantarius* ssp. *infantarius/S. lutetiensis*	7
		*S. thermophilus*	3
*S. gallolyticus* ssp. *macedonicus*	15	*S. gallolyticus* ssp. *macedonicus*	15
*L. lactis*	7	*L. lactis*	6
		N.I.	1
*S. thermophilus*	7	*S. thermophilus*	7
*E. faecium*	2	*E. faecium*	2
*E. faecalis*	2	*E. faecalis*	2

Abbreviation: N.I., non‐identified.

## 4 Discussion

Consumers' preferences are determined by features like the taste, aroma, and texture of the cheese. These properties may change depending on the chemical qualities of the raw milk, microbial flora, enzyme activity, and the storage conditions of cheese (Wilkinson and Kilcawley 2005). Besides the chemical quality and microbial flora of the raw milk, the metabolic reactions taking place during the maturation of the cheese, like proteolysis and lipolysis, influence the desired properties of the cheese. As a result of these metabolic reactions, volatile aromatic compounds such as short‐chained fatty acids, acetaldehyde, diacetyl, and free amino acids are synthesized (Singh et al. 2003; Awad et al. 2007). The instability of microflora in raw milk leads to imbalances in aroma compound concentrations and structure flaws during the maturation period of the cheese (Youssef et al. 2019). In this study, 15 Mihaliç cheeses out of 53 were selected based on their relatively higher organoleptic and physico‐chemical properties to determine and isolate appropriate lactic flora adapted to the specific ripening conditions of this cheese.

The chemical composition of Mihaliç cheese is one of the contributing factors to improving its organoleptic qualities. These chemical qualities include dry matter, protein, fat, pH, acidity and salinity. There are a limited number of studies on Mihaliç cheese's chemical composition. According to Aday and Karagul Yuceer (2014) dry matter, fat and protein percentages in Mihaliç cheese were 56.7%–64.1%; 25.2%–33%; and 18.6%–24.6%, respectively. The same study calculated pH, lactic acid percentage, and salt content as 5.09–5.99, 0.37–1.04, and 3.27–8.18, respectively. In another study, dry matter, fat, protein, pH, lactic acid, and salt were reported as 64.13, 24.58, 23.15, 5.86, 0.53, and 8.7, respectively (Özcan and Kurdal 2012). Generally, these results are consistent, but specific differences would be explained by factors such as production method, type and quality of the milk used, and the maturation level (Erceyes et al. 2018).

The salinity, the organic acids, the low aw value and the competing flora in the cheese environment are not suitable for the growth of most bacteria (Koch et al. 2010; Bove et al. 2012; Fox, Guinee, Cogan, and McSweeney 2017; Fox, Guinee, Cogan, McSweeney, Fox, et al. 2017). The salt tolerance of LAB cheese displays a difference. Generally, lactobacilli have the highest salinity tolerance (> 6%), followed by lactococci (4.5%–5%) and then *S. thermophilus* (< 3%), which has the lowest salinity tolerance (Johnson 2014). Streptococci show variations in environmental stress factors such as salinity, pH, and heat in general (Parente et al. 2014). Notably, the Streptococcaceae count in SMC samples was 1 log higher than those of lactobacilli and enterococci. Moreover, in the crust part over again, the count of the Streptococcaceae was almost 2 logs higher compared to lactobacilli and enterococci (Table 3). These results evidenced that the Streptococcaceae are the predominant LAB species in Mihaliç. Nevertheless, it should be kept in mind that enterococci and some other microorganisms can also grow on M17 agar (İrkin 2017; Gonzalez et al. 2017). There was also a significant difference in the counts of lactobacilli and enterococci between the crust and the inner parts of SMC and LSMC (*p* < 0.05), indicating that salt content in brine solution and metabolites formed during ripening might slow down the growth of some species in these groups.

The microbial enzymes that are important in the maturation of the cheese are the exo‐enzymes and endo‐enzymes like peptidase, aminotransferase, and lipase that are released upon cell autolysis (McSweeney et al. 1993; Collins et al. 2002). The enzymatic activities of LAB are essential in cheese maturation, particularly when the bacteria count reaches 10^6^–10^7^ CFU/g, which leads to the formation of taste and aroma compounds (Wilkinson and Kilcawley 2005; Fox, Guinee, Cogan, McSweeney, Fox, et al. 2017). Of the studies performed on Mihaliç cheese to determine the microorganism counts, Şen (1991) reports *Lactococcus* spp. count in raw‐milk Mihalic cheese as 3.74–5.23 log CFU/g, while the *Lactobacillus* spp. count was 1.82–3.67 log CFU/g. Accordingly, Özdemir et al. (2004) reported the LAB count as 5.41 log CFU/g. In our study, the lactobacilli and streptococci counts in SMC and LSMC samples were higher than both other researchers' findings and are sufficient for the development of the taste and aroma compounds for the cheese (Fox, Guinee, Cogan, and McSweeney 2017).

The results of this study revealed that most of the isolates were identified consistently at the species level by MALDI‐TOF MS, indicating that the technique could be used as a powerful tool for fast and reliable identification. Nevertheless, some genetically close species, such as *Lcb. paracasei*, *Lcp. casei* and *Lcb. rhamnosus* or *S. infantarius* ssp. *infantarius* and *S. lutesiensis*, which have similarities in ribosomal proteins, could not be differentiated by MALDI‐TOF MS. More precise and distinctive identification techniques are required to discriminate such closely related species/sub‐species (Quintela‐Baluja et al. 2013). Meanwhile, since classical identification techniques and sequencing are more time‐consuming and require relatively higher costs, MALDI‐TOF MS could be employed as a fast, economical, robust and reliable method for the characterization of LAB species on routine analysis.

Like most artisanal cheeses produced without starter cultures, *Lactococcus* spp. were the predominant bacteria. *L. lactis* is used as the principal acid‐producing starter culture in many cheeses (Mills et al. 2007; Buyukyoruk et al. 2010; Cibik et al. 2010). It is isolated from raw milk, bovine skin, and certain plants and takes place in the flora of many non‐pasteurized cheeses (Klijn et al. 1995). It is responsible not only for acidification but also for the formation of aromatic compounds during cheese maturation. With their endo‐enzymes liberated as a consequence of autolysis, lactococci participate in aromatic compounds such as α‐ketoglutarate (Gómez de Cadiñanos et al. 2018).

Interestingly, we determined *L. lactis* together with *S. gallolyticus* ssp. *macedonicus* as the other predominant streptococci in the inner and crust parts of both SMC and LSMC. Other streptococci, *S. thermophilus* and *S. infantarius* ssp. *infantarius* were also identified, but to a lesser extent. Strikingly, *S. infantarius* ssp. *infantarius* and *S. gallolyticus* ssp. *macedonicus* classified in the *S. bovis/S. equinus* complex (SBSEC) were isolated from various traditionally produced cheeses over the last decade (Tsakalidou et al. 1998; Schlegel et al. 2003; Demir and Kaptan 2025). Nevertheless, it is well evidenced that some members of SBSEC such as *S. gallolyticus* ssp. *gallolyticus* and *S. gallolyticus* ssp. *pasteurianus* are responsible for endocarditis, bloodstream infection, and colorectal cancer (Boleij and Tjalsma 2013; Chang et al. 2023; Abdelgadir et al. 2008; Jans et al. 2013). The role and possible contribution of this species in cheese maturation need to be studied in detail. Due to the fact that some species within the SBSEC group are involved in the aforementioned clinical health problems, detailed studies are needed to reveal the survival and pathogenicity of the members of this group in dairy products.

*Lim. fermentum* and *Lcb. paracasei* followed by *Lcb. rhamnosus* were the most isolated lactobacilli species in both crust and inner parts. *L. delbrueckii* spp., *Lat. curvatus* and *Lbp. plantarum* were also present in the flora of Mihaliç. The use of lactobacilli as starters or adjuncts for cheese ripening or as probiotic cultures in various foods has been increasing. To produce enhanced flavors during cheese ripening, their recommended counts should be higher than 10^4^cfu/mL (Peterson and Marshall 1990). For proper fermentation, artisanal cheeses made without adding any starter culture depend on raw milk's endogenous homofermentative and heterofermentative LAB, including *Lcb. paracasei, Lpb. plantarum, Lim. fermentum, L. delbrueckii*, and *Levilactobacillus brevis*, which are generally considered NSLAB (Di Cagno et al. 2007; Oberg et al. 2022). In the inner part of Mihaliç, the obligate heterofermentative LAB *Lim. fermentum* was predominant compared to other lactobacilli, while *Lcb. paracasei* was relatively predominant in the crust part. Compared to the other dairy lactobacilli, its esterolytic activity is higher and could contribute to the fruity flavors of cheeses. The desired fruity taste in the inner part of Mihaliç might be derived from the activity of *Lim. fermentum*. Moreover, the CO_2_ formed by the heterofermentative degradation of lactose could also be responsible for the formation of several eyes in the inner part of Mihaliç. As a member of the NSLAB population, *Lim. fermentum* is one of the most abundant species in natural whey starters for Caciocavallo Silano, a hard ‘pasta filata’ cheese, and Grand Padano cheese (Rossetti et al. 2008). It is also frequently part of certain cheeses, including Swiss Comtè (Cremonesi et al. 2011), Ragusano (Randazzo et al. 2002), and Manchego (Sánchez et al. 2006).

Other LAB species identified in Mihaliç are homofermentative *Lcb. paracasei*/*casei* and closely related *Lcb. rhamnosus* (formerly *Lactobacillus casei* group). It is commonly used in dairy fermentation as adjunct cultures and has probiotic properties contributing to health promotion. They were present both in the crust and inner parts of SMC and LSMC but are predominant in the crust part, suggesting they are more resistant to salinity and environmental conditions. Minervini and Calasso (2022) proved that they are more suited to cheese environments and less inhibited by salt, pH, and environmental conditions. They can also grow over 10°C–40°C, but some strains can grow at 5°C and some up to 45°C (Minervini and Calasso 2022). On the other hand, there is an adversary growth between *Lbc. paracasei* and *Lim. fermentum* in dairy products. Laleye et al. (1989) reported that homofermentative such as *Lcb. casei, Lcb. paracasei*, and *Lactiplantibacillus plantarum* can inhibit the growth of heterofermentative lactobacilli such as *Levilactobacillus brevis* and *Lim. fermentum*.

*Enterococcus* spp. also are frequently isolated from artisanal cheeses and may contribute to the development of aromatic components due to their proteolytic and lipolytic enzymes (Centeno et al. 1996; Arizcun et al. 1997; Cogan et al. 1997; Foulquie Moreno et al. 2006). They were proposed as NSLAB cultures in cheese technology and have promising probiotic and anti‐bacterial potential (Benmouna et al. 2024); however, these microorganisms are opportunistic and responsible for the development of antibiotic resistance among the bacteria (Suzzi et al. 2000; Giraffa 2003; Özmen Toğay et al. 2016). Like most other artisanal cheeses made from raw milk, *E. faecium* and *E. faecalis* were the predominant enterococci in Mihaliç cheese. They were isolated from the crust and inner part due to their persistence and growth ability at relatively high salt (Martinez‐Murcia and Collins 1991).

The results of the present work evidenced that *Lim. fermentum* and *Lcb. paracasei/casei* in particular and to a lesser extent *Lcb. rhamnosus* and *Lpb. plantarum* might be potential lactobacilli which may be used as starters in Mihaliç once their technological properties are determined. Furthermore, *L. lactis* are predominantly isolated from Mihaliç cheese, which indicates their possible role in ripening. Interestingly, like many other artisanal cheeses tested over the last decades, *S. infantarius* ssp. *infantarius* and *S. gallolyticus* ssp. m*acedonicus* species were apparently well adapted to the Mihalic environment and isolated predominantly; nevertheless, their potential needs to be scrutinized since they may represent potential public health risks due to their opportunistic pathogenic properties.

## 5 Conclusion

The microbial composition of Mihaliç cheese revealed distinct variations depending on salting conditions (SMC vs. LSMC) and spatial distribution (crust vs. inner parts). Streptococcaceae demonstrated strong adaptation to the maturation environment, particularly in the crust, while lactobacilli and enterococci were more sensitive to salinity, showing higher prevalence in the inner parts and in LSMC samples. *Lcb. casei/paracasei* and *Lim. fermentum* were the predominant lactobacilli. Similarly, *L. lactis* was identified as the major species, while *Streptococcus gallolyticus ssp. macedonicus* and *Str. infantarius* ssp. *infantarius* further contributed to the characteristic flora, reflecting the artisanal and raw milk origin of the cheese. *E. faecium* and *E. faecalis* were the major species isolated. Overall, the findings highlight the complex and diverse lactic flora of Mihaliç cheese, underlining the key role of microbial adaptation to salinity and ripening environment in shaping its unique microbial ecosystem.

## Author Contributions

**Ergün Ayanoğlu:** writing – review and editing, writing – original draft, methodology, investigation. **Hakan Tavşanlı:** conceptualization, methodology, supervision, writing – review and editing. **Recep Cibik:** methodology, writing – review and editing.

## Funding

The authors receive no specific funding.

## Conflicts of Interest

The authors declare no conflicts of interest.

## Data Availability

Data will be made available on request.
